# Abnormal functional connectivity of the frontostriatal circuits in type 2 diabetes mellitus

**DOI:** 10.3389/fnagi.2022.1055172

**Published:** 2023-01-04

**Authors:** Yingxia Fu, Meiling Gu, Rui Wang, Juan Xu, Shenglu Sun, Huifeng Zhang, Dejian Huang, Zongjun Zhang, Fei Peng, Pan Lin

**Affiliations:** ^1^Affiliated Hospital of Integrated Traditional Chinese and Western Medicine, Nanjing University of Chinese Medicine, Nanjing, China; ^2^Jiangsu Province Academy of Traditional Chinese Medicine, Nanjing, China; ^3^Department of Psychology, Nanjing Normal University, Nanjing, China; ^4^Department of Psychology and Cognition and Human Behavior Key Laboratory of Hunan Province, Hunan Normal University, Hunan, China

**Keywords:** type 2 diabetes mellitus, frontostriatal circuits, functional connectivity, resting-state, functional magnetic resonance imaging

## Abstract

**Background:**

Type 2 diabetes mellitus (T2DM) is a metabolic disorder associated with an increased incidence of cognitive and emotional disorders. Previous studies have indicated that the frontostriatal circuits play a significant role in brain disorders. However, few studies have investigated functional connectivity (FC) abnormalities in the frontostriatal circuits in T2DM.

**Objective:**

We aimed to investigate the abnormal functional connectivity (FC) of the frontostriatal circuits in patients with T2DM and to explore the relationship between abnormal FC and diabetes-related variables.

**Methods:**

Twenty-seven patients with T2DM were selected as the patient group, and 27 healthy peoples were selected as the healthy controls (HCs). The two groups were matched for age and sex. In addition, all subjects underwent resting-state functional magnetic resonance imaging (rs-fMRI) and neuropsychological evaluation. Seed-based FC analyses were performed by placing six bilateral pairs of seeds within *a priori* defined subdivisions of the striatum. The functional connection strength of subdivisions of the striatum was compared between the two groups and correlated with each clinical variable.

**Results:**

Patients with T2DM showed abnormalities in the FC of the frontostriatal circuits. Our findings show significantly reduced FC between the right caudate nucleus and left precentral gyrus (LPCG) in the patients with T2DM compared to the HCs. The FC between the prefrontal cortex (left inferior frontal gyrus, left frontal pole, right frontal pole, and right middle frontal gyrus) and the right caudate nucleus has a significant positive correlation with fasting blood glucose (FBG).

**Conclusion:**

The results showed abnormal FC of the frontostriatal circuits in T2DM patients, which might provide a new direction to investigate the neuropathological mechanisms of T2DM.

## 1. Introduction

Type 2 diabetes mellitus (T2DM) is a metabolic disease with an increasing incidence worldwide (Lees et al., 2009; Bobbert et al., 2013). This disease constitutes a risk factor associated with an increased incidence of cognitive dysfunctions and emotional disorders [e.g., Parkinson’s disease (PD), Alzheimer’s disease (AD), obsessive–compulsive disorder (OCD), and major depressive disorder (MDD)] (den Braber et al., 2010; Pan et al., 2010; Zhang et al., 2013; Ashraghi et al., 2016; Athauda and Foltynie, 2016; De Pablo-Fernandez et al., 2018; Cheong et al., 2020; Wei et al., 2020; Sánchez-Gómez et al., 2021; Fanelli et al., 2022). However, the specific neural substrate of T2DM-related cognitive impairment and emotional disorders remains unclear. The frontostriatal circuits have been shown to play an important role in the pathophysiology of a variety of cognitive and emotional disorders (Casey et al., 2007; Baggio et al., 2015; Achterberg et al., 2016; Gramespacher et al., 2020; Tomiyama et al., 2022). Given the impairment of cognitive and emotional functioning in T2DM, the role of frontostriatal connectivity in T2DM is attracting considerable attention (Looi, 2017; Pérez-Taboada et al., 2020).

The frontostriatal circuits form a looped structure wherein information is delivered directly from the frontal cortex to the striatum, while the frontal cortex receives information from major outputs of the striatum indirectly *via* the thalamus (Gopinath et al., 2011; Mandelbaum et al., 2019). Different cortical projection areas can form different circuits with the basal ganglia: sensorimotor circuits involve premotor cortical projections; associative circuits involve the dorsolateral prefrontal and parietal cortex; limbic circuits primarily involve the orbitofrontal and medial prefrontal cortices. These circuits contribute to motor control, motor learning, emotions and association (Lanciego et al., 2012; Barber et al., 2019; Young et al., 2022). Disrupted connectivity of these circuits may underlie certain abnormalities that occur in various cognitive and emotional disorders (Kwak et al., 2010; Posner et al., 2014; Huang et al., 2015; Tomiyama et al., 2021). Recently, an increasing number of studies have provided significant insight through neuroimaging of frontostriatal circuits.

Resting-state functional magnetic resonance imaging (rs-fMRI) is a promising approach with which to studying the brain-related pathophysiology of T2DM (Chau et al., 2021; Lei et al., 2022). Functional connectivity (FC) is an effective method to explore impaired frontostriatal circuits (Di Martino et al., 2008; Kelly et al., 2009). Previous rs-fMRI studies have indicated that abnormal FC of the frontostriatal circuits impacts cognitive functions and emotional regulation (Casey et al., 2007; Achterberg et al., 2016; Lin et al., 2018; Gramespacher et al., 2020; Tomiyama et al., 2022). For example, several studies have revealed that frontostriatal connectivity is reduced in patients with PD and AD (Middei et al., 2004; Anderkova et al., 2017; Nieuwhof et al., 2017). Moreover, disruption of FC within reward-relevant corticostriatal neurocircuitry is associated with reduced motivation and motor slowing in depression (Felger et al., 2016). More importantly, previous research has shown that T2DM has complex associations with cognitive and emotional disorders; for example, T2DM increases susceptibility to PD, AD, MDD, and more (D’Amelio et al., 2009; Kumar et al., 2014; Zhang et al., 2017; Cheong et al., 2020). In AD, an increased risk of cognitive decline among diabetic patients is well documented (Kodl and Seaquist, 2008; Reijmer et al., 2010; Rawlings et al., 2014). Regarding PD, a number of epidemiological studies indicate that the incidence is increased in people with preexisting diabetes (Cereda et al., 2011; Xu et al., 2011; Yang et al., 2017). However, the mechanisms of this relationship are unknown. Previous studies have shown that insulin resistance is associated with disease progression, increased severity of dyskinesia, and increased risk in patients with PD (Bosco et al., 2012; Athauda and Foltynie, 2016). Furthermore, a number of patients with T2DM in the absence of PD exhibit pathologies related to subclinical striatal dopaminergic dysfunction (Cheong et al., 2020), and T2DM and PD share common etiopathogenic mechanisms (Santiago and Potashkin, 2014). We speculate that there are possible alterations in frontostriatal circuit connectivity in this population.

Some recent studies have provided further relevant evidence. A study using fMRI reported that FC in the frontostriatal circuit was decreased in patients with type 1 diabetes mellitus compared to normal controls (Croosu et al., 2022). More importantly, multiple studies have shown structural abnormalities of the frontostriatal circuit in T2DM. For example, the magnetization transfer ratio (MTR) representing the biophysical integrity of the cortico-striato-pallido-thalamic circuits was compromised in patients with T2DM even in the absence of significant cognitive impairments or mood disturbances (Yang et al., 2015). In addition, meta-analyses to date have demonstrated a significant decrease in gray matter volume (GMV) in the cortico-striato-limbic network in patients with T2DM (Wu et al., 2017). This finding further demonstrated the important role of the frontostriatal circuitry in the pathogenesis of T2DM. T2DM-related changes in these circuits may contribute, in part, to increased susceptibility to cognitive deficits and/or emotional disorders in patients with T2DM and potentially indicate the underlying substrates linking T2DM and cognitive and emotional disorders.

However, to the best of our knowledge, there have been no studies of dysfunctional frontostriatal connectivity in T2DM. In this study, we hypothesized that abnormalities in the FC of the frontostriatal circuitry would be associated with T2DM. To test this hypothesis, we performed rs-fMRI analyses to examine the FC of the frontostriatal circuitry in patients with T2DM and HCs. More importantly, we investigated the relationship between abnormal FC of the frontostriatal circuitry and the clinical indicators of T2DM.

## 2. Materials and methods

### 2.1. Participants

The medical research ethics committee of Jiangsu Integrated Traditional Chinese and Western Medicine Hospital examined this cross-sectional research plan and granted ethical approval. A total of 31 patients who met the latest diagnostic criteria for T2DM (American Diabetes Association, 2013) were recruited from the Endocrinology Department of Jiangsu Integrated Traditional Chinese and Western Medicine Hospital, and 31 healthy controls (HCs) were recruited through advertisements. The T2DM group and the HC group were matched for age, sex, and education level. All subjects participated voluntarily and signed informed consent forms. Because some patients had incomplete clinical data or imaging data, only 27 patients with T2DM and 27 patients with HCs were ultimately included. Table 1 presents the demographic and clinical characteristics of all participants. All subjects were right-handed individuals between 30 and 75 years of age and had no contraindications to MRI. The patients had a diabetes duration of at least 1 year and no complications. They were closely self-monitored and routinely treated with various hypoglycemic agents, and none had any history of hypoglycemic episodes. The exclusion criteria for both groups were as follows: (1) abnormal brain structure, including the presence of tumors or a history of trauma, surgery, or cerebrovascular accidents; (2) neurological or mental disorders, such as depression, dementia, schizophrenia or epilepsy.

**Table 1:** Demographic and clinical characteristics with T2DM and healthy controls.

Characteristics	T2DM patients (*n* = 27)	HCs (*n* = 27)	*p*-values
Age (years)	55.03 ± 10.8	54.56 ± 9.79	0.751
Sex (male/female)	14/13	11/16	1.000
Weight(kg)	65.93 ± 9.86	65.96 ± 13.54	0.796
HbA1c(%) mmol/mol)	9.04 ± 2.44	5.42 ± 0.72	0.000
Fasting glucose (mmol/L)	10.69 ± 3.47	5.33 ± 0.86	0.000
Cognitive performance			
MMSE	26.44 ± 2.29	27.96 ± 1.76	0.009
Directional force	9.85 ± 0.36	9.92 ± 0.27	0.396
Auditory verbal memory test	2.93 ± 0.27	2.96 ± 0.46	0.561
Attention and computing power	3.414 ± 1.55	3.83 ± 1.44	0.101
Auditory verbal memory test-delay	2.44 ± 0.75	2.74 ± 0.45	0.156
Language power	8.11 ± 1.34	8.59 ± 0.67	0.123
MoCA	21.15 ± 3.9	23.41 ± 3.99	0.042
Alternate wiring test	0.296 ± 0.47	0.741 ± 0.45	0.001
visual structural skills1 (cube)	0.407 ± 0.5	0.59 ± 0.5	0.180
visual structural skills2 (clock)	2.15 ± 0.91	2.48 ± 0.7	0.137
Name	2.82 ± 0.48	2.7 ± 0.61	0.461
Memory(No points)	/	/	——
Attention	4.62 ± 1.39	5.48 ± 0.85	0.06
Repeat	1.29 ± 0.72	1.52 ± 0.58	0.219
Word fluency	0.7 ± 0.8	0.44 ± 0.58	0.299
Abstract force	0.96 ± 0.89	1.0 ± 0.87	0.879
Delayed recall	1.67 ± 1.49	2.67 ± 1.66	0.024
Orientation force	5.85 ± 0.36	5.93 ± 0.27	0.391

### 2.2. Clinical and neuropsychological data

The history and clinical data of patients with T2DM were obtained from medical records and questionnaires. The clinical data of HCs, along with demographic data such as gender and age, were obtained through a questionnaire. After overnight fasting, blood samples were obtained to measure the levels of fasting blood glucose (FBG) and glycosylated hemoglobin (HbA1c). The Mini-Mental State Examination (MMSE) and Montreal Cognitive Assessment (MoCA) were used to assess the general cognitive function of diabetes patients and HCs.

### 2.3. Image acquisition

A 3.0 TMRI (Discovery 750) scanner from GE (United States) was used for MRI examination. During imaging, subjects wore eye masks and were instructed to remain awake and keep their heads still. Soft pads were used to reduce head movement, and headphones were used to reduce noise. rs-fMRI scans were obtained by using a gradient-recalled echo echo-planar imaging (GRE-EPI) sequence (repetition time (TR) =2,000 ms; echo time (TE) =30 ms; flip angle (FA) = 90 degrees; layer spacing =0 mm; layer thickness = 4 mm; field of view (FOV) =220 mm × 220 mm; matrix dimensions = 64 × 64. A fast gradient-echo sequence (magnetization-prepared rapid gradient echo, MPRAGE) was used to obtain high-resolution 3D T1-weighted structure images. The acquisition parameters were as follows: TR = 8.2 ms, TE = 3.2 ms, inversion time (TI) = 900 ms, FA = 9°, FOV = 256 mm × 256 mm, and voxel size = 1.3 mm × 0.9 mm × 5 mm; these parameters were used for image registration and functional positioning.

### 2.4. Data processing

Rs-fMRI and structural images were analyzed using the CONN toolbox (Version 18.a). Preprocessing of fMRI and structural images was performed using CONN’s default preprocessing pipeline. The preprocessing pipeline included functional realignment and unwarping, slice-timing correction, outlier identification, segmentation, normalization of functional and anatomical images to the standard Montreal National Institute (MNI) template, outlier rejection, and functional smoothing. The data were smoothed spatially with an 8-mm full width at half maximum Gaussian kernel. The fMRI data were further denoised by using the component-based noise correction method (CompCor) to remove signal contributions from brain white matter, cerebrospinal fluid, and motion parameters. Finally, a temporal bandpass filter of 0.008–0.09 Hz was applied, and linear detrending was performed.

### 2.5. FC analysis

**Figure 1:**
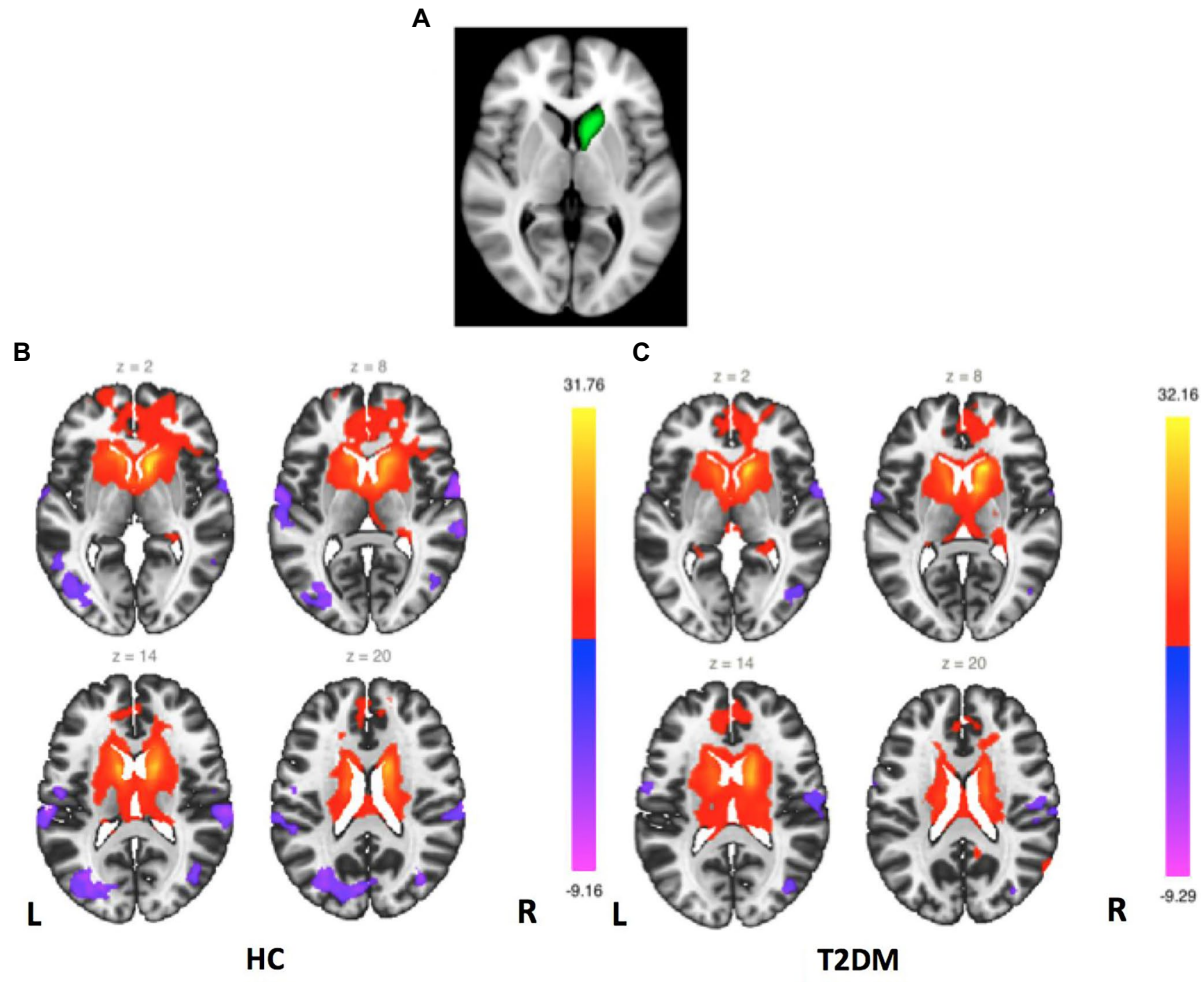
Right caudate FC maps. **(A)** Right caudate seed region. **(B)** FC maps for the HC group (second-level analysis, *p* < 0.05, FDR corrected). **(C)** FC maps for the T2DM group (second-level analysis, *p* < 0.05, FDR corrected).

For seed-based FC analyses, six ROIs—the bilateral caudate, bilateral putamen and bilateral pallidum—were created according to anatomical parcellation atlases included in the CONN toolbox (Harvard-Oxford Probabilistic Atlas, see Figure 1). For the first-level analyses, the average BOLD time series of each seed was correlated with remaining voxels in the brain, and FC connectivity maps were derived by Fisher’s r-to-z transformation to carry out second-level analyses. For the second-level analyses, within-and between-group analysis of results from the T2DM and HC groups was further performed. For within-group comparisons, a whole-brain false discovery rate (FDR)–corrected threshold of *p* < 0.05 was used to identify areas of significant functional connectivity, and between-group analyses were performed to compare FC changes using two-sample t tests. The results of the seed-based analyses are reported at the following thresholds: uncorrected voxelwise *p* < 0.001 and cluster-level familywise error (FWE)-corrected *p*-FWE <0.05. In addition, we investigated the relationship between clinical symptoms and the FC maps of the right caudate seed region. Group-level analysis of variance was conducted, in which the clinical indicators were used as covariates to identify the association between the resting-state FC index and T2DM. In this covariate analysis, correlations between the FC and clinical scores were considered significant at a cluster-corrected *p*-FWE <0.05.

### 2.6. Statistical analysis

We used SPSS 21.0 to analyze differences in demographic and clinical data as well as neuropsychological scores between the T2DM patients and the HCs. The mean ± standard deviation (x ®± s) and percentage (%) are used to express numerical and categorical data, respectively. The chi-square test was used to compare the gender distributions of the two groups; Kolmogorov–Smirnov tests were performed for each group to verify the normality of other numerical data distributions. Depending on whether the distributions were normal or nonnormal, two-independent-sample t tests and Mann–Whitney U tests were used to identify significant differences between the T2DM and HC groups, *p* < 0.05 was considered statistically significant.

## 3. Results

### 3.1. Clinical and neuropsychological data

The clinical characteristics and neuropsychological data of the patients with T2DM and the HCs are summarized in Table 1. No significant group differences were observed in age, sex, or weight (all *p* values >0.05). However, the patients exhibited significantly higher levels of HbA1c and FBG than the HCs (all p values <0.05). The patients also performed significantly worse on two neuropsychological tasks, namely, the Alternate Wiring Test and delayed recall (*p* < 0.05), which mainly involve the cognitive domains of processing speed, executive function and episodic memory (Table 1).

### 3.2. FC of the striatum sub-regions

**Figure 2:**
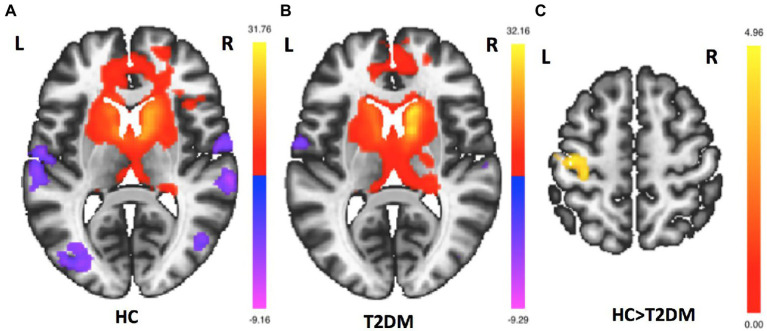
Group differences in right caudate FC maps. **(A)**. Right caudate FC maps for the HC group (*p* < 0.05, FDR–corrected). **(B)**. Right caudate FC maps for the T2DM group (*p* < 0.05, FDR–corrected). **(C)** Group differences in right caudate FC between the HC and T2DM groups in voxelwise whole-brain analysis. Compared to HCs, T2DM patients had reduced FC between the right caudate and left precentral gyrus (*p* < 0.05, FWE corrected).

FC mappings generated with striatum sub-regions as the seeds were remarkably consistent with previous studies (Supplementary Figures S1–S6). Figure 1 shows the right caudate FC maps of the HC and T2DM groups. Only significant differences in the FC of the right caudate were found between these two groups (*p* < 0.05, FWE corrected). As shown in Figure 2, significantly reduced FC was observed between the right caudate and the left precentral gyrus in the T2DM patients compared to the HCs. No significant differences in the FC of the left caudate, bilateral putamen and bilateral pallidum were found between these two groups.

### 3.3. Correlations between fasting glucose and right caudate FC

**Figure 3:**
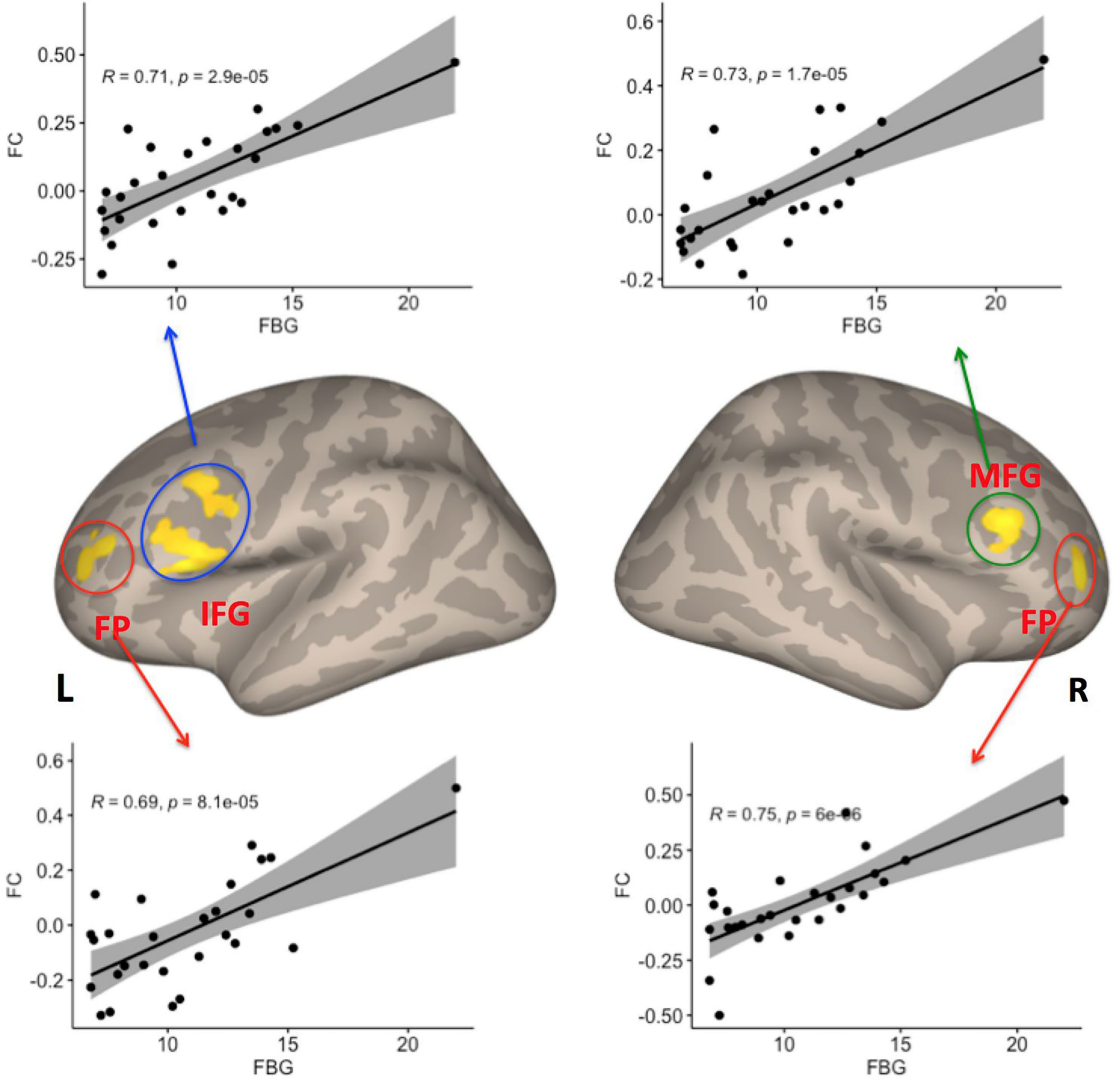
Four clusters showed significant correlations between FC with the right caudate and fasting blood glucose (FBG) variables among patients with T2DM (*p* < 0.05, FDR–corrected). IFG, inferior frontal gyrus; FP, frontal pole; MFG, middle frontal gyrus; FDR, false discovery rate, L Left; R Right.

To test our hypothesis regarding an association between the severity of T2DM variables and right caudate connectivity abnormalities in the T2DM group, we performed a correlation analysis. We found that FBG was significantly positively correlated with the connectivity between right caudate and the LIFG, LFP, RFP and RMFG (see Table 2; Figure 3).

**Table 2:** Correlations with FBG variables in T2DM patients.

**Brain region**	**MNI (*x*, *y*, *z*)**	**Cluster size**	**Tmax**	***p* FDR**
Left IFG	−38,22,16	312	7.50	0.000060
Left FP	−36,54,14	99	5.01	0.026387
Right MFG	42,26,24	85	5.60	0.036207
Right FP	38,62,10	102	5.36	0.026387

IFG, inferior frontal gyrus; FP, frontal pole; MFG, middle frontal gyrus; FDR, false discovery rate; MNI, Montreal Neurological Institute.

## 4. Discussions

In this study, we used rs-fMRI to investigate the frontostriatal circuits in patients with T2DM compared with HCs. We found abnormal FC in the frontostriatal circuits within T2DM patients. The FC between the right caudate nucleus and the left precentral gyrus (LPCG) was significantly reduced in patients with T2DM. Moreover, the FC between the prefrontal cortex (left inferior frontal gyrus, or LIFG; left frontal pole, or LFP; right frontal pole, or RFP; and right middle frontal gyrus, or RMFG) and the right caudate nucleus had a significant positive correlation with FBG. This result suggests that the frontostriatal circuits are involved in the neuropathology of cognitive impairment in T2DM, which might provide a new direction for research into the neuropathological mechanisms of T2DM.

### 4.1. Alterations in the FC of prefronto-striatal sensorimotor loops in T2DM

Consistent with previously reported findings (Franzmeier and Dyrba, 2017), our study found that the FC between the right caudate nucleus and the LPCG was significantly reduced in patients with T2DM. The frontostriatal sensorimotor loops play an important role in sensorimotor control. Many studies have found that the striatum may contribute to habit formation and motor control by continuously integrating task-relevant information to constrain the execution of motor habits (Albin et al., 1989; Rueda-Orozco and Robbe, 2015; Sales-Carbonell et al., 2018). The caudate nucleus is a critical part of prefronto-striatal loops that have been consistently implicated in behavior (McColgan et al., 2015; Ji et al., 2018). The PCG is the site of the premotor cortex, which mainly manages skeletal muscle movements throughout the body; at the same time, some proprioceptive fibers also project to the PCG to modulate somatosensation (Christensen et al., 2007; Marotta et al., 2021). Previous studies have shown that patients with T2DM have dyskinesias and sensory disturbances. For example, T2DM is associated with decreased muscle strength, postural instability, and gait difficulties (Park et al., 2006, 2009; Kotagal et al., 2013; Cheong et al., 2020). Furthermore, the peripheral neuropathy associated with T2DM may lead to sensory impairments that compromise the function of the motor system (Allen et al., 2016). Thus, deficits in prefrontal-striatal sensorimotor loops between the right caudate nucleus and LPCG may be the neuropathological basis of motor and sensory dysfunction in T2DM.

A number of studies have examined the correlations of FC in frontostriatal sensorimotor loops with movement-related disorders. For example, a previous rs-fMRI study of MDD reported that the frontostriatal sensorimotor loops are involved in psychomotor changes, such as hypo-and hyperactivity (Schmaal et al., 2016). More importantly, structural neuroimaging studies have demonstrated biophysical abnormalities in the head of the caudate nucleus in patients with T2DM and MDD. fMRI studies probing motor inhibition in PD have shown that the frontostriatal systems are functionally impaired (Schernhammer et al., 2011; Kumar et al., 2014; Qin et al., 2021). Thus, our studies support the existence of an association between preexisting T2DM and movement-related disorders (e.g., MDD and PD) at the level of brain function.

However, the pathophysiological mechanism underlying the abnormal FC of prefronto-striatal sensorimotor loops in T2DM has remained unclear. Some studies indicate that changes in brain function are associated with changes in brain structure (Espeland et al., 2013; Karama et al., 2014). Previous studies have found that high glucose and insulin resistance in T2DM promote striatal oxidative stress, alter dopamine neurotransmission, and increase the chance of nigrostriatal neuron damage (Morris et al., 2011; Pérez-Taboada et al., 2020). Another previous study revealed that T2DM was associated with the loss of subcortical gray matter in the caudate nucleus and putamen (Moran et al., 2013). Thus, the damage to striatal structures may be responsible for the FC changes in the frontostriatal sensorimotor loops in the T2DM group.

### 4.2. Effects of hyperglycemia on the FC of prefronto-striatal circuits in T2DM

A number of studies have reported relationships between FBG and the FC of brain networks or regions in T2DM. For example, some studies have reported that reduced FC is associated with an increase in FBG (Xia et al., 2013; Chen et al., 2014; Liu et al., 2016). In contrast, one study reported that fasting plasma glucose was positively correlated with the FC between the left fusiform gyrus and the right MFG (Guo et al., 2021). In line with this previous study, our present study also found a positive correlation between FBG and the FC between the right caudate nucleus and LIFG, LFP, RFP, and RMFG in patients with T2DM.

The LIFG, LFP, and RMFG are components of the prefrontal cortex, which plays a key role in feeding control, food craving, and metabolic regulation (Li et al., 2013; Gluck et al., 2017). The caudate nucleus is a critical part of the striatum and is involved in mediating hunger and satiety (Holsen et al., 2012), food reward, food-seeking behaviors, and food-related emotion-regulatory memory processes (Schoenbaum et al., 1998; Miller, 2000; Petrovich et al., 2002; Saper et al., 2002). Accordingly, the prefronto-striatal circuits have been demonstrated to exhibit altered FC in organisms with altered feeding behavior. For example, some studies have reported that participants with obesity displayed increased FC between the ventral striatum and the medial prefrontal cortex, which are linked to food craving and weight gain (Contreras-Rodríguez et al., 2017; Contreras-Rodriguez et al., 2019) Our findings may further support the notion that the prefronto-striatal circuit plays an important role in feeding behavior. Abnormal feeding behavior (e.g., excessive eating) is one of the typical clinical symptoms of T2DM and is closely linked to the epidemic of obesity (DeFronzo et al., 2015; Leitner et al., 2017). One possibility is that the FC of prefronto-striatal circuit enhancement may reflect the activation of neural circuits governing food reward in T2DM patients. Furthermore, T2DM is an energy utilization disorder in which blood sugar cannot be converted into energy, resulting in hunger that significantly increases activation in the reward-salience circuitry (ventral striatum, dorsal caudate, anterior cingulate cortex) during the processing of immediate reward (Wierenga et al., 2015), which may be the neural basis for the clinical symptoms of abnormal feeding behavior in T2DM.

Overall, T2DM is a chronic metabolic disease that is closely related to many unhealthy lifestyle habits, including obesity caused by excessive eating. Abnormal FC of the prefronto-striatal circuit may contribute to a reduced ability to control food intake and abnormal eating habits in T2DM, leading to metabolicdisorder.

## 5. Limitations

This study has some limitations. First, this study had a cross-sectional design and a small sample size, which may have resulted in limited statistical power. We will perform an additional study to confirm the present findings in patients with T2DM. Second, the indicators used in the clinical evaluation were not comprehensive; further studies should add insulin indicators, Motor function, body mass index (BMI), and food addiction (FA) scores. Third, the Increased FBG possibility caused by the FC of prefronto-striatal circuit enhancement to activate the neural circuits governing food reward in T2DM patients, However, we did not measure relevant clinical indicators, We should add food addiction (FA) scores in future studies. Finally, although we demonstrated altered FC of the frontostriatal circuitry in T2DM, its contribution to behavioral and cognitive impairments in T2DM is not fully understood. Further studies should incorporate cognitive tasks and motor function assessments to investigate cognitive and motor dysfunction arising from the frontostriatal circuitry in T2DM.

## 6. Conclusion

In conclusion, this rs-fMRI study revealed abnormal frontostriatal FC in patients with T2DM. Moreover, this abnormality was closely related to blood glucose, which expands the understanding provided by previous studies and further supports the concept that the frontostriatal circuits play an important role in the pathogenesis of T2DM. Our findings provide important insights into the pathogenetic processes of T2DM.

## Data availability statement

The raw data supporting the conclusions of this article will be made available by the authors, without undue reservation.

## Ethics statement

The studies involving human participants were reviewed and approved by the Medical Research Ethics Committee of Jiangsu Integrated Traditional Chinese and Western Medicine Hospital. The patients/participants provided their written informed consent to participate in this study.

## Author contributions

YF and FP: drafted the manuscript and designed the study. DH, ZZ, and PL: helped to design the study and revised the manuscript. YF, MG, and PL: performed the statistical analysis. RW and SS: provided technical support. JX and HZ: collected the clinical data. All authors contributed to the article and approved the submitted version.

## Funding

This work was supported in part by the National Natural Science Foundation of China (No. 62071177).

## Conflict of interest

The authors declare that the research was conducted in the absence of any commercial or financial relationships that could be construed as a potential conflict of interest.

## Publisher’s note

All claims expressed in this article are solely those of the authors and do not necessarily represent those of their affiliated organizations, or those of the publisher, the editors and the reviewers. Any product that may be evaluated in this article, or claim that may be made by its manufacturer, is not guaranteed or endorsed by the publisher.
