# Supplementary material for: Abnormal functional connectivity of the frontostriatal circuits in type 2 diabetes mellitus
Source: Front Aging Neurosci. 2023 Jan 4;14:1055172. doi: 10.3389/fnagi.2022.1055172 (PMC9846649; doi:10.3389/fnagi.2022.1055172)
Supplement: Supplementary file 1 [file Data_Sheet_1.PDF]

Supplementary Materials

Abnormal functional connectivity of the frontostriatal circuits in type 2 diabetes mellitus

Fig. S1. Functional connectivity maps of the left caudate. (A) Right caudate seed region. (B) FC maps for the HC group (second-level analysis, $p<0.05$, FDR corrected). (C) FC maps for the T2DM group (second-level analysis, $p<0.05$, FDR corrected).

Fig. S2 . Functional connectivity maps of the right caudate. (A) Right caudate seed region. (B) FC maps for the HC group (second-level analysis, $p<0.05$, FDR corrected). (C) FC maps for the T2DM group (second-level analysis, $p<0.05$, FDR corrected).

Fig. S3 . Functional connectivity maps of the left putamen. (A) Right caudate seed region. (B) FC maps for the HC group (second-level analysis, $p<0.05$, FDR corrected). (C) FC maps for the T2DM group (second-level analysis, $p<0.05$, FDR corrected).

Fig. S4 . Functional connectivity maps of the right putamen. (A) Right caudate seed region. (B) FC maps for the HC group (second-level analysis, $p<0.05$, FDR corrected). (C) FC maps for the T2DM group (second-level analysis, $p<0.05$, FDR corrected).

Fig. S5 . Functional connectivity maps of the left Pallidum. (A) Right caudate seed region. (B) FC maps for the HC group (second-level analysis, $p<0.05$, FDR corrected). (C) FC maps for the T2DM group (second-level analysis, $p<0.05$, FDR corrected).

Fig. S6 . Functional connectivity maps of the right pallidum. (A) Right caudate seed region. (B) FC maps for the HC group (second-level analysis, $p<0.05$, FDR corrected). (C) FC maps for the T2DM group (second-level analysis, $p<0.05$, FDR corrected).

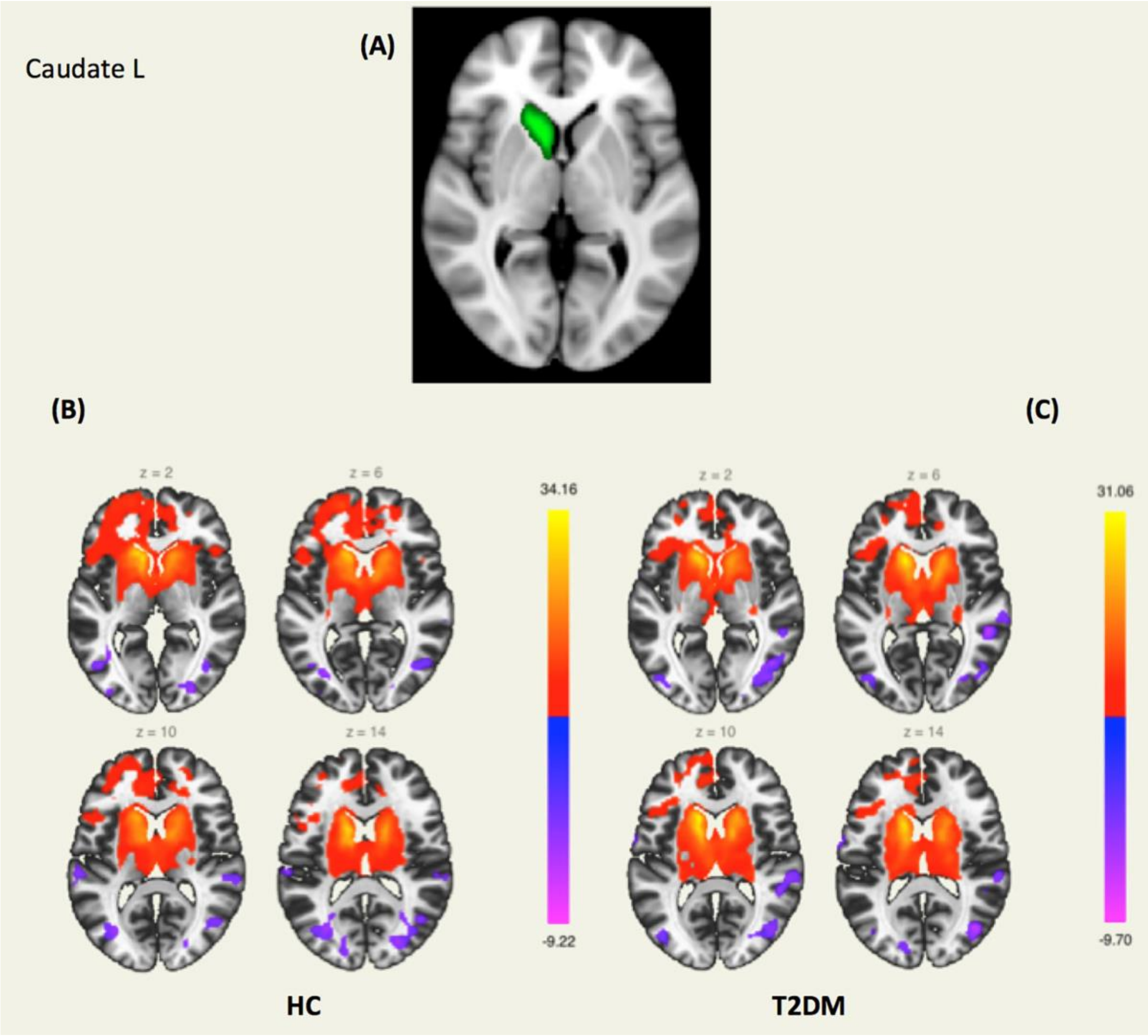

Fig. S1 Functional connectivity maps of the left caudate. (A) Right caudate seed region. (B) FC maps for the HC group (second-level analysis, $p<0.05$, FDR corrected). (C) FC maps for the T2DM group (second-level analysis, $p<0.05$, FDR corrected).

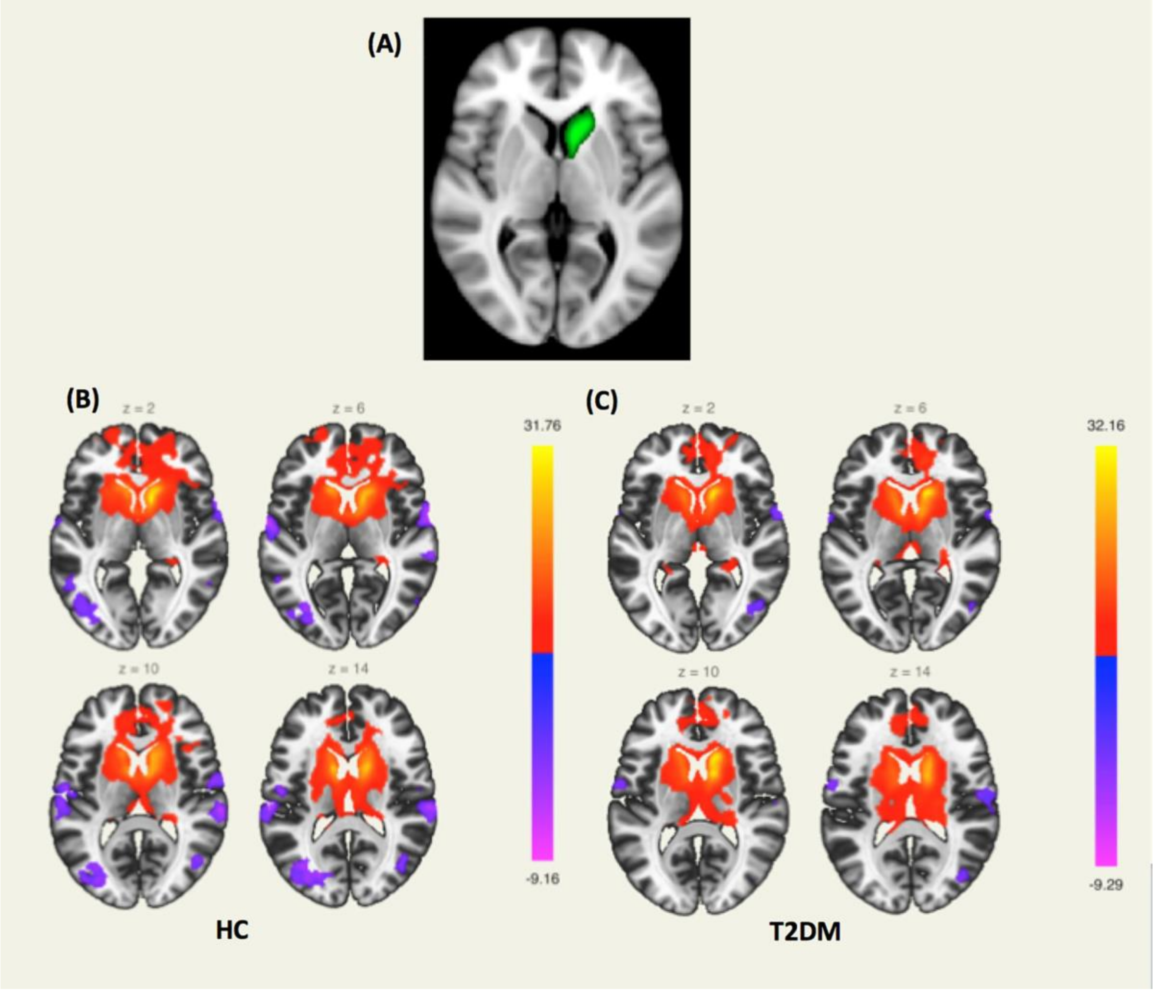

Fig. S2 Functional connectivity maps of the right caudate. (A) Right caudate seed region. (B) FC maps for the HC group (second-level analysis, p<0.05, FDR corrected). (C) FC maps for the T2DM group (second-level analysis, p<0.05, FDR corrected).

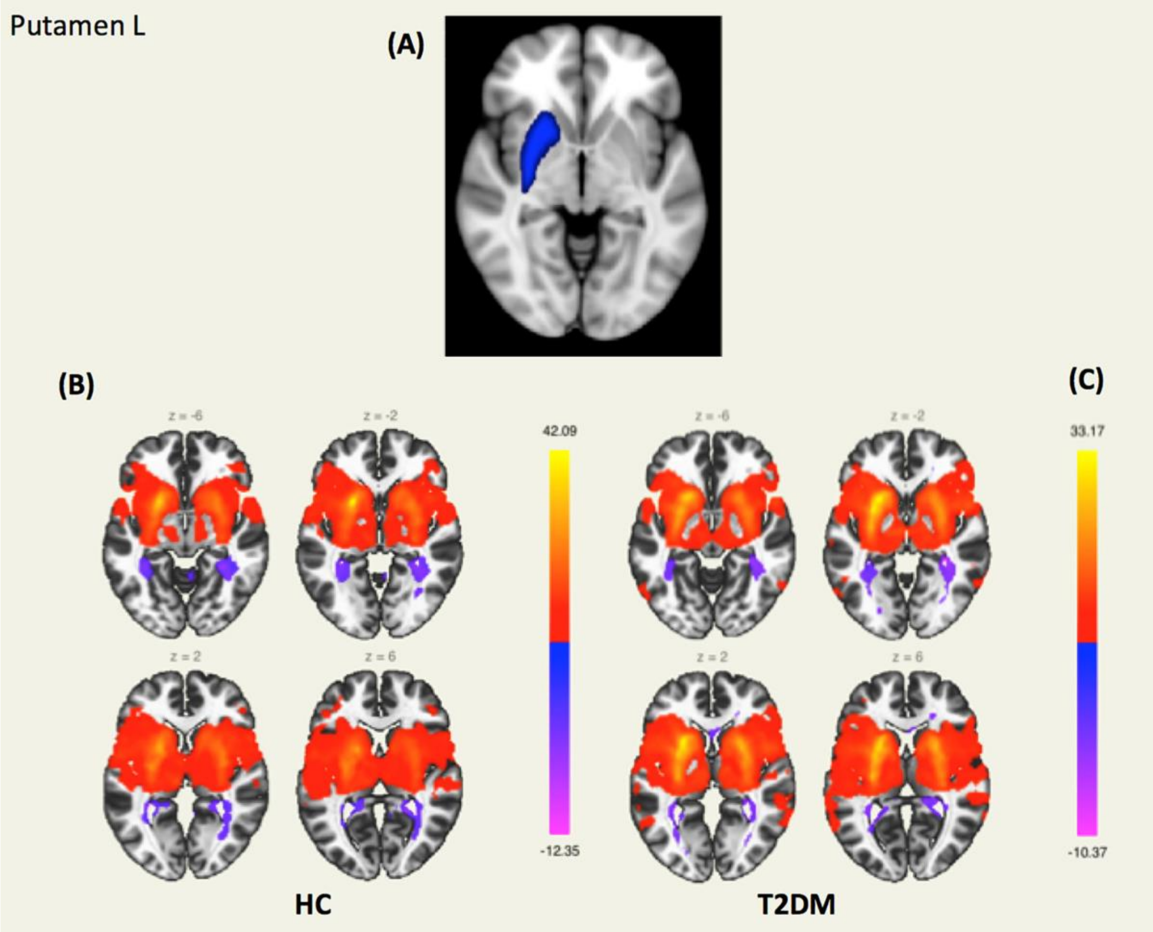

Fig. S3 Functional connectivity maps of the left putamen. (A) Right caudate seed region. (B) FC maps for the HC group (second-level analysis, $p<0.05$, FDR corrected). (C) FC maps for the T2DM group (second-level analysis, $p<0.05$, FDR corrected).

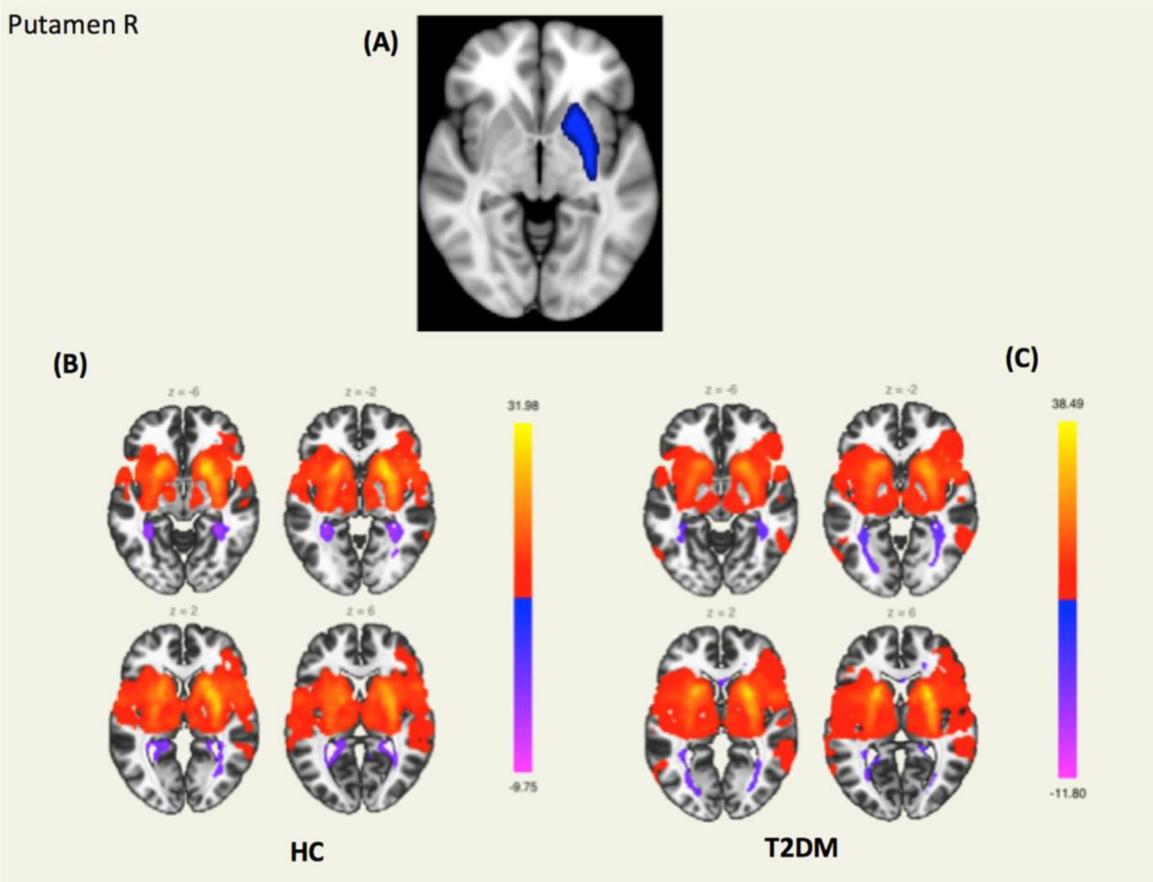

Fig. S4 Functional connectivity maps of the right putamen. (A) Right caudate seed region. (B) FC maps for the HC group (second-level analysis, p<0.05, FDR corrected). (C) FC maps for the T2DM group (second-level analysis, p<0.05, FDR corrected).

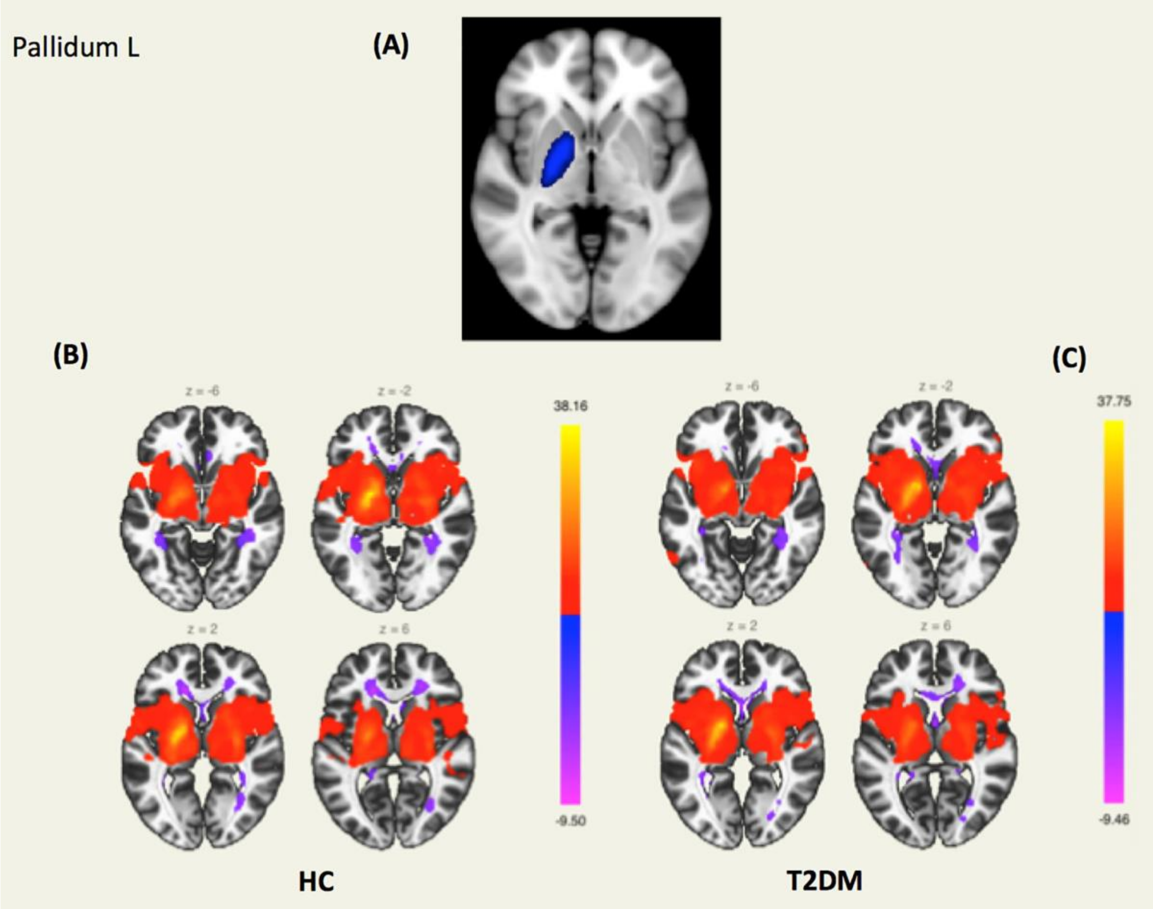

Fig. S5 . Functional connectivity maps of the left pallidum. (A) Right caudate seed region. (B) FC maps for the HC group (second-level analysis, p<0.05, FDR corrected). (C) FC maps for the T2DM group (second-level analysis, p<0.05, FDR corrected).

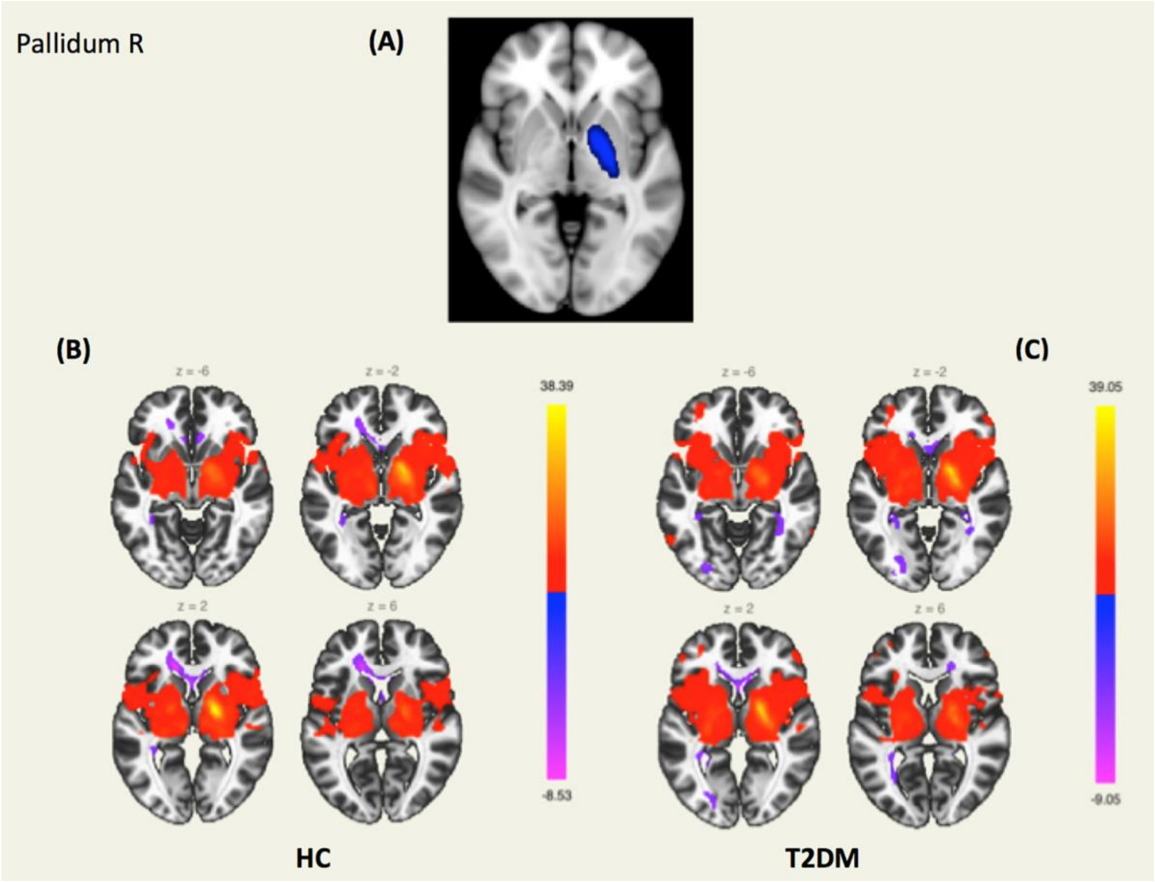

Fig. S6 . Functional connectivity maps of the right pallidum. (A) Right caudate seed region. (B) FC maps for the HC group (second-level analysis, $p<0.05$, FDR corrected). (C) FC maps for the T2DM group (second-level analysis, $p<0.05$, FDR corrected).
